# Photodynamic Anti-Bacteria by Carbon Dots and Their Nano-Composites

**DOI:** 10.3390/ph15040487

**Published:** 2022-04-18

**Authors:** Xiaoyan Wu, Khurram Abbas, Yuxiang Yang, Zijian Li, Antonio Claudio Tedesco, Hong Bi

**Affiliations:** 1 School of Chemistry and Chemical Engineering, Anhui University, Hefei 230601, China; wuxiaoyan1213@163.com (X.W.); abbaskhurram93@gmail.com (K.A.); yyx18715093366@163.com (Y.Y.); atedesco@usp.br (A.C.T.); 2 School of Materials Science and Engineering, Anhui University, Hefei 230601, China; 22018@ahu.edu.cn; 3 Department of Chemistry, Center of Nanotechnology and Tissue Engineering-Photobiology and Photomedicine Research Group, Faculty of Philosophy, Sciences and Letters of Ribeirão Preto, University of São Paulo, Ribeirão Preto, São Paulo 14040-901, Brazil

**Keywords:** carbon dots, antimicrobial, light activation, photodynamic effect, reactive oxygen species

## Abstract

The misuse of many types of broad-spectrum antibiotics leads to increased antimicrobial resistance. As a result, the development of a novel antibacterial agent is essential. Photodynamic antimicrobial chemotherapy (PACT) is becoming more popular due to its advantages in eliminating drug-resistant strains and providing broad-spectrum antibacterial resistance. Carbon dots (CDs), zero-dimensional nanomaterials with diameters smaller than 10 nm, offer a green and cost-effective alternative to PACT photosensitizers. This article reviewed the synthesis methods of antibacterial CDs as well as the recent progress of CDs and their nanocomposites in photodynamic sterilization, focusing on maximizing the bactericidal impact of CDs photosensitizers. This review establishes the base for future CDs development in the PACT field.

## 1. Introduction

Infections caused by fungi, bacteria, parasites, or viruses cause many severe diseases. Our healthcare systems face substantial problems, from treatment needs to prevention in hospital settings and routine work dealing with many critical pathologies, food and water environments and sources protection, and worldwide public health impact [1,2]. Antibiotics have historically been the primary weapon in the fight against infectious diseases. However, due to the high cost and long pathways to new drugs discovery, clinical testing, and scaling up the production process, approval of the development of next-generation antibiotics takes longer [3]. Additionally, bacteria have several ways of rapidly acquiring resistance, which can endanger the health of patients and delay wound recovery after treatment [4]. As a result of multidrug resistance (MDR), many of these diseases will become more challenging to treat and result in higher medical costs and mortality rates [5,6,7]. Since the appearance of multidrug resistance in pathogenic bacteria, traditional antibiotics/antimicrobials cannot meet the expectations of today’s society and the urgent needs to efficiently prevent and treat a considerable spectrum of bacterial infections [8]. It is imperative to find and develop alternative antibacterial techniques to combat MDR effectively and to prevent and treat diseases and their undesirable side effects. In environmental contamination, the consequences and expenses to eliminate the impact could be worse and could take a year and in some cases decades [9].

Photodynamic inactivation of bacteria mediated by photoactive compounds, more precisely photosensitizer molecules (PSs), is one of the most promising techniques in the fight against MDR pathogens [10], such that as used and developed in remote ancient Egypt approximately 4000 years ago, when a skin disease such as vitiligo was treated by a combination of plants orally administered and exposure of the patients to sunlight. The successes of the treatment were a result of photodynamic reactions mediated by a natural product present in the extract of *Ammimajus*, a furanocoumarin and a psoralen. Photodynamic antimicrobial chemotherapy (PACT) is a fast, intense and challenging field that has been developed to address the growing antibiotic resistance among harmful bacteria [11]. It was developed in response to the need for better treatment and prevention of bacterial infectious diseases.

Several issues are involved in PACT, including the design and choice of the nanostructured photoactive molecules and their isolation or synthetic route to make it feasible to penetrate the cellular cytoplasm or induce specific damage to the cellular organelles in the target tissue [12]. Fungi are eukaryotic microorganisms, similar to mammalian cells, and the development of new antifungal drugs remains challenging due to a number of reasons, such as the presence of the nucleus and the structure of the cell wall. Bacteria are prokaryote microorganisms that can be easily distinguished from mammalian cells. Conversely, fungal diseases are usually caused by pathogens (fungi), and these fungal diseases feature a variety of symptoms that are commonly related to the attack of the skin and respiratory systems. Fungi and bacteria can form biofilms versus staying in their planktonic forms, increasing their drug resistance [13].

Carbon dots (CDs) have been proposed as a potential fluorescent nanomaterial for identifying and inactivating different types of bacterial species among a wide variety of PSs, already used in the past [14,15]. CDs are carbon-based nanomaterials that are quasi-spherical in shape and have a typical size of less than 10 nm. They have good photoelectric properties, high water solubility, and chemical durability. CDs also present low toxicity and have good biocompatibility, making them ideal for bioimaging [16,17,18,19,20], drug delivery [21], gene delivery [22], biosensors [21] and fluorescent-labeling applications [23,24]. CDs are well known to undergo optical absorption via π-plasmon transitions [9]. In contrast, fluorescence emission occurs in the visible to the near-infrared spectral range due to photogenerated holes and electrons trapped at different surface sites and associated radiative recombination [25]. CDs exhibit powerful photodynamic effects due to their optical properties [26], which have been exploited to kill bacterial and cancer cells under visible light irradiation [27].

In this review, we summarize the most common synthetic methods for producing CDs and CDs nanocomposites, their application in photodynamic antimicrobial applications in recent years, and the factors and improvements affecting the antimicrobial effectiveness of CDs.

## 2. Synthesis Techniques of Carbon Dots Employed for Antimicrobials

The properties of CDs are closely related to their preparation methods [28]. Top-down and bottom-up approaches are two commonly used approaches for preparing CDs [29,30]. Carbon quantum dots’ final physicochemical and functional properties, including their photophysical behaviors, biocompatibility and antibacterial activity, are influenced by the method employed and the carbon source used during the synthetic process [31].

Using a top-down technique, large-sized carbon materials, such as carbon nanotubes and graphite ash, are decomposed into small CDs, from the macro to the nanoscale. Different carbon sources are exposed to laser ablation, arc discharge, plasma treatment, chemical oxidation, electrochemical oxidation, and others [32,33,34,35,36]. Using different types of acid treatment, the concentration of the oxygen-containing groups attached to the CDs structure can be easily changed. However, doping additional materials onto CDs is tricky and is a powerful option to potentialize the CDs’ nanomaterial. Furthermore, the strong acid may cause CDs to lose their conjugated structure, changing their photophysical properties, resulting in lower absorption and emission wavelengths [37].

Chemical processes such as hydrothermal, pyrolysis, combustion, ultrasonic, microwave irradiation, thermal, and biogenic procedures, conversely, are used in the bottom-up approach [38,39]. CDs can also be prepared using non-graphite carbon sources such as tiny polymers and monomers as carbon precursors [40,41]. Using this method, a wide range of elements, including N, P, S, B, and even metal ions, can be doped into the CDs’ structure [42]. The addition of heteroatoms to the CDs structure improves the fluorescent properties of these nanomaterials by changing the absorption and emission peak positions and boosting the fluorescent quantum yield. Wu et al. found a link between CDs’ photo-oxidation activity, phosphorescent quantum yield, and N content, underlining the importance of N-doping in boosting CDs’ photosensitization performance [43]. Marković et al. found that the photodynamic antibacterial properties directly impact the ROS production by the CDs doping process with F and Cl compared with undoped nanoparticles [44]. It is essential to optimize the photodynamic antibacterial effect of CDs by choosing suitable precursors and the proper selection of doping elements. However, doping sites and better concentrations are still challenging to manage and archive.

Unfortunately, many of the current methods require toxic chemicals and solvents, high temperatures, long reaction times and complex processing steps. Therefore, the development of green chemistry concepts to manufacturing fluorescent CDs through simple, economical and sustainable pathways represents a meaningful topic [45]. More recently, efforts have been devoted to utilization of green carbon sources and the development of green synthesis processes. For the former, biomass, which is renewable organic material that comes from plants and animals, represents a typical green carbon source. The use of biomass for CDs synthesis, especially in large-scale production, is attracting increasing attention among researchers. For the green synthesis process, toxic chemicals that are harmful to people’s health and the environment should be avoided. In addition, the preparation procedure, reaction time and conditions should be optimized to increase economic efficiency. Currently, the main green synthesis processes for producing CDs include ultrasonication, microwave irradiation, hydrothermal carbonization, self-exothermic synthesis, and ozone/hydrogen peroxide oxidation [46].

## 3. Carbon Dots in Antimicrobial Photodynamic Therapy

**Figure 1:**
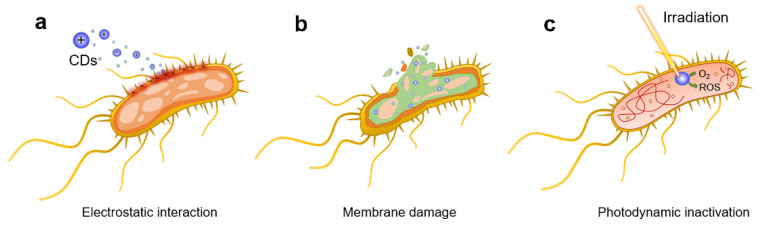
General bactericidal mechanisms of action of CDs. (**a**) Schematic representation of the initial electrostatic interaction between CDs and the bacterial cell wall. (**b**) CDs internalization, intercalation in the bacterial membrane, and irreversible disruption with a leak of cytoplasmatic material. (**c**) CDs-promoted bacterial photodynamic inactivation with ROS production and DNA damage. Reproduced with permission from Ref. [10]. Copyright © 2021 *Nanomaterials* MDPI.

The currently known antibacterial mechanism of CDs is shown in Figure 1. CDs with positively charged surfaces interact electrostatically with negatively charged bacteria, facilitating CDs internalization and killing bacteria [47,48] This CDs behavior is critical for the success of the PACT. As observed in the past, bacteria and biofilm frameworks present a tremendous challenge in the treatment protocol design, considering the difficulty of the photoactive compounds penetrating through the exopolysaccharide matrix [49]. Neutral or negatively charged photoactive compounds have been proposed to treat Gram-negative bacteria without success. The low permeability of the Gram-negative outer membrane avoids effective incorporation of molecules, reducing the light activation effect. Previous examples of treatment of the tissue with biological or chemical agents, such as CaCl_2_ or Tris-EDTA, which are expected to increase the release of the molecules by up to 50% of the outer membrane lipopolysaccharide present as desired, but they showed undesirable side effects in a clinical trial [50,51].

Bacteria can also be killed by disintegrating bacterial cell walls, resulting in cytoplasmic material leaking [52], which could induce secondary side effects. Furthermore, the higher temperature caused by the photothermal therapy (PTT) effect or the release of ROS [53] by the PACT effect can directly damage the bacterial DNA and proteins, leading to a bluster effect. CDs are also valued for their capacity to produce highly active ROS. CDs’ present a visible and near-infrared spectrum of light absorbance, destroying bacteria through classical mechanisms’ photoinduced production of ROS.

### 3.1. Photosensitization Mechanisms

**Figure 2:**
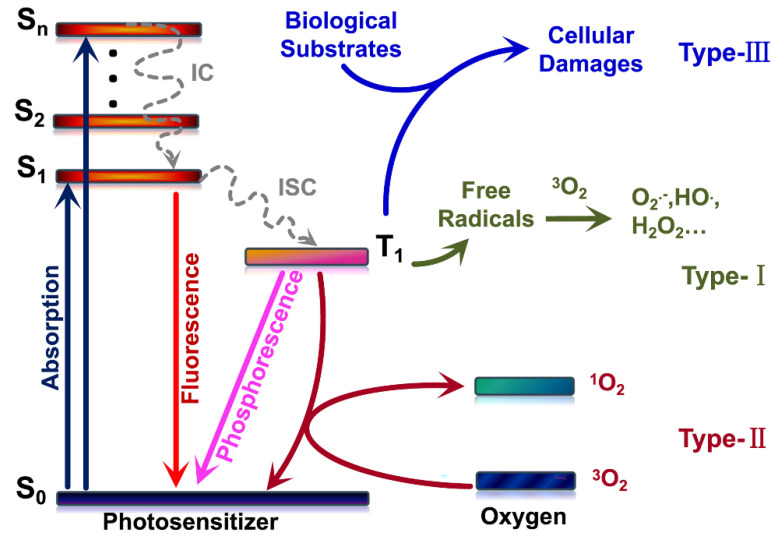
Schematic illustration of three types of mechanisms in PDT. Reproduced with permission from Ref. [59]. Copyright © 2022 *Chem* Elsevier B.V.

PDT was discovered in the 20th century, has received much attention, and has established a background in cancer treatment worldwide because of its advantages, such as fewer side effects, less invasive surgery, and repeatable treatment [54,55,56]. It has also been used as adjunctive therapy and works synergistically with the classical approaches for cancer. The classical photophysical and photochemical steps involved in PDT are summarized by Jablonski Diagram. Basically, the PSs in the ground non-excited state (S_0_) can absorb visible light (photons), moving to the first electronically excited singlet state (S_1_), and then by intersystem crossing (ISC), produce, by spin inversion, the first excited triplet state (T_1_). The excited triplet state (T_1_) has a long lifetime (around milliseconds)—enough to move forward by energy and electron transfer processes. The deactivation of the first excited state was sometimes a fast and helpful process, defined by a fluorescence emission used in many cases for diagnostic. The excited triplet state (T_1_) deactivation is a phosphorescence process with long-time light emission. It can also be deactivated by a thermal decay that can also be used to disperse the energy of the excited PS. Three classical photoreductions govern their photoreaction on PDT and photon energy transfer mechanisms (Type I, Type II, and Type III (Figure 2)) found today [57,58,59]. Photoactive compounds acting by the Type I mechanism are primarily based on hydrogen atoms or electrons that transfer from the excited photosensitizer molecules *(T1) states, react with oxygen, and produce ROS products such as HO^•^, O_2_^•−^, and H_2_O_2_ [60,61,62,63].

Photosensitization mechanism Type I-Redox reactions (1–4) with biomolecules [64].

(1) S0→S1→S3

(2) S3+Sub→Sub+•+Sub-• (electron transfer),

(3) S−•+O32→Sub+O2•−→HO•+HO•

S = photosensitizer molecule and Sub = organic substrate.(4)

Conversely, photoactive compounds operating by Type II are propelled by electron spin exchange between the photosensitizer *(T_1_) and triplet oxygen (^3^O_2_), which results in the T_1_–T_1_ annihilation process and the formation of non-radical but highly reactive singlet oxygen (^1^O_2_). 

Mechanism photosensitizing Type II-mediated production ^1^O_2_, such as lipid peroxidation, is based on the above reaction (1) [64] and then follows reactions (5–6).

(5) S3+O32→S0+O12 (energy transfer),

(6) O12+Sub → Sub-OOH(peroxides, etc.),

The reactive oxygen species produced by the photosensitization Type I mechanism are constantly made in the living organisms at a low level [65,66,67]. Since these species are highly reactive, the microorganisms need protective systems to neutralize the action of these radicals. This protective system includes enzymes such as superoxide dismutase (SOD) that have its activity over the superoxide anion radical, as well as the catalase and the peroxidase, which control the harmful effects of hydrogen peroxide:

(7) $$O_{2}^{•-}{+O}_{2}^{•-}{+2H}^{+}\underset{\to }{\;\mathrm{SOD}\;}H_{2}O_{2}$$

(8) $${2H}_{2}O_{2}\underset{\to }{\;\mathrm{catalase}\;}H_{2}{O+O}_{2}$$

(9) $$H_{2}O_{2}{+2H}^{+}\underset{\to }{\;\mathrm{peroxidase}\;}{2H}_{2}O^{\;}$$

In the Type II mechanism, energy is transferred directly to the molecular oxygen. The PSs return to the ground state after an absorption cycle and active oxygen generation. The photodynamic effect is directly affected by the single-linear oxygen yield in the Type II mechanism [68,69,70,71].

PSs should also be selectively targeted biomolecules such as nucleic acids, proteins, and other macromolecules by a Type III mechanism. When paired with Type III photosensitizers, the PSs can directly and effectively destroy the exciting biological target molecule [59].

Few PSs can be employed in Type III PDT due to the stringent requirements for photosensitizers. CDs have not yet been proven to work by Type III PDT but remain an open challenge. Type II PDT may be the most studied of the three mechanisms, and ^1^O_2_ QY is a valuable metric of photophysical properties for assessing photodynamic performance [72,73]. The better the photodynamic effect, the higher the ^1^O_2_ QY. Several methods for detecting ^1^O_2_ include electron spin resonance (ESR), fluorescence, UV–visible absorption indirect method (classical Donors–Acceptor quenching process) and by time-resolved methods such as flash photolysis and near-infrared (NIR) ^1^O_2_ detection. Fluorescence and UV–visible absorption can be used to calculate the QY of ^1^O_2_ [74,75].

### 3.2. Photodynamic Anti-Bacteria by Carbon Dots

PACT can damage DNA, oxidize amino acids, inactivate enzymes, and kill bacteria by releasing ROS. Because of its features of low cumulative toxicity, high spatial targeting processes, and drug resistance independence, PACT is considered a practical approach for antibacterial applications. Under visible and NIR light irradiation, exposed CDs produce ROS by electrons or by an energy transfer process, leading to ROS, employed as a new photosensitive nano-agent in PACT against microorganisms [76,77,78,79].

**Figure 3:**
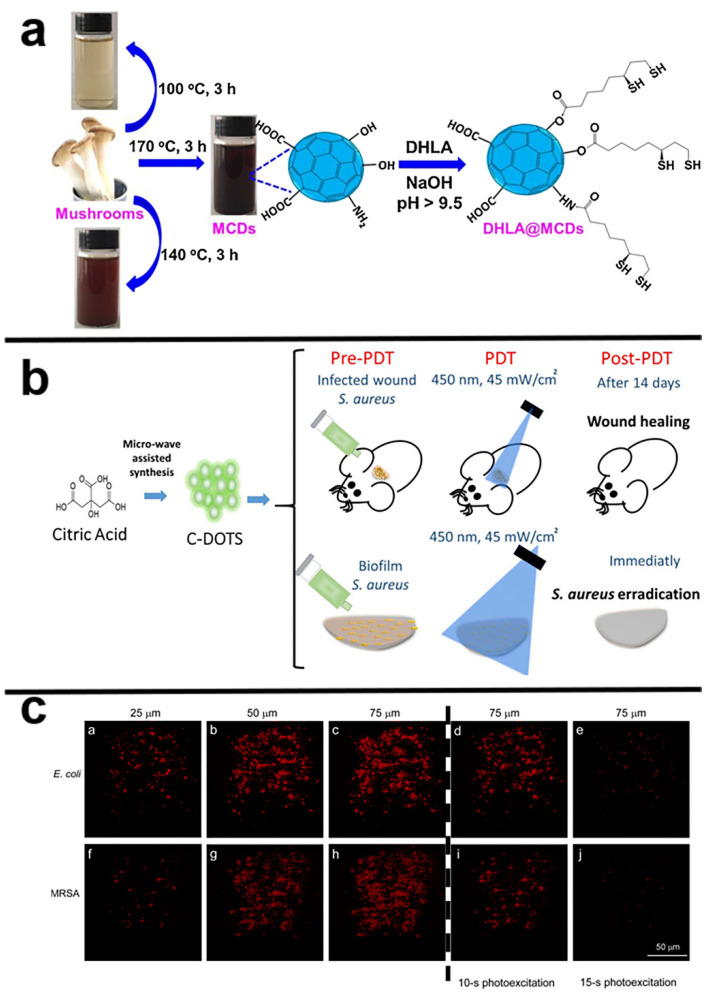
(**a**) Schematic illustration of synthesis of MCDs by thermal treatment at various temperatures and surface functionalization with DHLA. Reproduced with permission from Ref. [78]. Copyright © 2017, *Sensors and Actuators B: Chemical*, Elsevier B.V. (**b**) Brief description of the procedures carried out in this study. In vivo and in vitro PACT studies evaluated PACT mediated by CDs and blue LED light against *S. aureus*. Reproduced with permission from Ref. [80]. Copyright © 2021 *Frontiers in Microbiology*, Romero, Alves, Stringasci, Buzzá, Ciol, Inada and Bagnato. (**c**) Two-photon luminescence images of *E. coli* and MRSA Excitation wavelength: 800 nm. Delivered dose: OD_600_ ∼ 0.05 of bacteria and 0.50 μg mL^−1^ GQD-Ab_LPS_ and 0.50 μg mL^−1^ GQD-Ab_protein A_, respectively. Reproduced with permission from Ref. [83]. Copyright © 2022 *ACS Applied Materials & Interfaces*, American Chemical Society.

Some CDs, such as graphene [76] and mushroom CDs [78], have intrinsic photodynamic characteristics that do not require extra surface changes or doping processes. Trajkovic et al. used an electrochemical approach to make graphene quantum dots that can produce reactive oxygen species under photoexcitation (470 nm, 1 W) and kill Gram-negative bacteria such as *Escherichia coli* and methicillin-resistant *Staphylococcus aureus* (MRSA) [76]. As shown in Figure 3a, Yoon et al. used mushrooms as a raw material to make carbon dots (MCDs) with high blue fluorescence, with the most significant emission at 456 nm under the excitation of 360 nm UV light. Under LED, visible light illumination (2.70 mW cm^−2^), MCDs can produce ROS such as hydroxyl radicals and superoxide radicals, which can directly adhere to the surface of *Escherichia coli* (*E. coli*) cells and induce cell membrane damage [78]. Conversely, CDs have an effective photodynamic action with the advantages of photostability, non-toxicity, and high quantum yield of fluorescence, and use citric acid as the sole carbon source in the production of CDs. The entire process is considered a green and inexpensive environmental safety process and material production with many medical applications [78]. Bagnato et al. used citric acid as a raw material to prepare CDs that displayed an antibacterial photodynamic effect. It emits its maximum light at 530 nm when excited by light at 450 nm. CDs-mediated PACT removes *Staphylococcus aureus* (*S. aureus*) suspensions and biofilms, as shown in Figure 3b, and is an effective, affordable, and simple PACT reagent that may be utilized in both in vitro and in vivo studies [80].

However, UV–vis light can induce side effects and damage to the human body and has a low penetrability to tissue. Still, near-infrared light (NIR, 780–1700 nm) has an advantage in PACT because of its longer wavelength, less scattering, tissue absorption, and more importantly, higher penetration efficiency of biological tissue [81,82]. Pu et al. obtained graphene oxide (GO) sheets. As shown in Figure 3c, they prepared two-photon GQDs capable of producing ROS through ultrasonic shearing, with high two-photon absorption, a large two-photon excitation absolute cross-section (TPE), and two-photon solid luminescence of the NIR. It is possible to perform two-photon bioimaging and two-photon photodynamic therapy on both Gram-negative and Gram-positive bacterial [83].

Although some organic photosensitizers have a high singlet oxygen yield (i.e., rose benga (RB, 75%), methylene blue (MB, 52%), indocyanine green (ICG, 0.80%)) [55,84,85,86], CDs have outstanding advantages such as biocompatibility, water solubility, targeting specificity, NIR absorption, fast and reliable synthetic processes and many others features. Hence, CDs photosensitizers continue to have promising antibacterial uses. Some organic PSs, such as curcumin and riboflavin, have been under evaluation for decades and present remarkable photostability but poor water solubility, reducing their photodynamic effect [87,88]. Su et al. used a hydrothermal approach to create curcumin carbon dots (Cur-NRCDs) with imaging and antibacterial properties. They used curcumin (Cur), neutral red (NR), and citrate (CA) as raw materials. Under 405 nm excitation, Cur-NRCDs fluoresced brightly red. Cur-NRCDs had better photosensitivity than Cur. Cur-NRCDs have outstanding antibacterial activity, cytocompatibility, photostability, and ROS efficiency. Under xenon lamp irradiation, Cur-NRCDs can inactivate 100% *E. coli* and *S. aureus* at 15 and 10 mM concentrations, respectively [88].

Several investigations have been conducted in recent years to improve the antibacterial efficacy of CDs photosensitizers [9]. As previously stated, increasing the nitrogen concentration in CDs helps to strengthen the PACT effect. Kuo et al. prepared graphene oxide sheets using a modified Hummers process and produced N-GQDs using an ultrasonic shear reaction method. N-GQDs have a bright PL emission spectrum in the near-infrared region, at 728 nm. Their superior luminescence properties and photostability make them a viable contrast agent for bacteria tracking in bioimaging techniques. Under 670 nm (0.10 W cm^−2^) laser irradiation, a considerable amount of ^1^O_2_ and O_2_^•−^ may be created simultaneously, and the effect of killing *E. coli* can approach 100% efficacy in only three minutes of light exposure. They also discovered that components with higher nitrogen content in graphene quantum dots could perform photodynamic therapy more effectively after the same treatment than components with lower nitrogen bonding content, indicating that future clinical applications, particularly for multidrug-resistant bacteria, are possible [89]. Probably, the mechanism of action works by synergic production of ROS and RNO (reactive nitrogen species).

**Figure 4:**
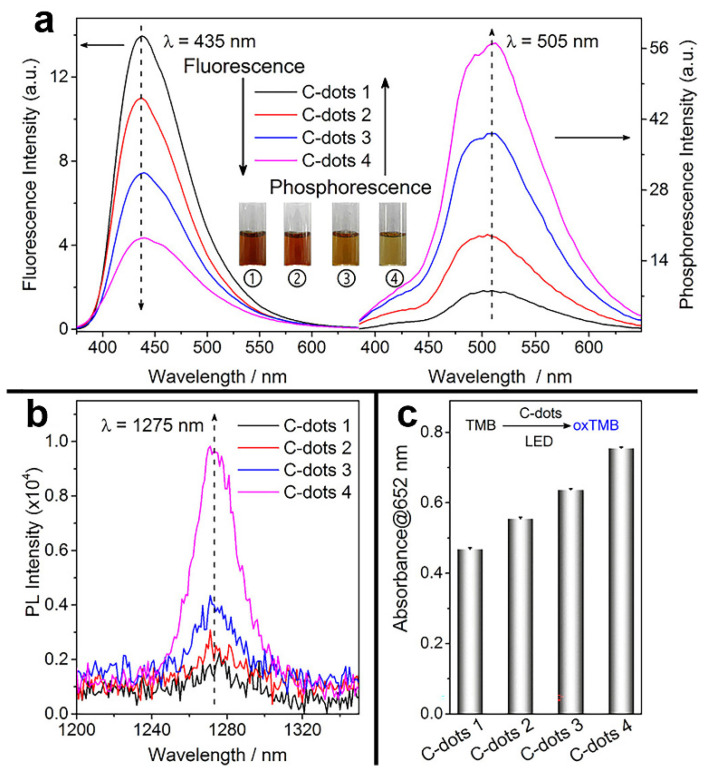
Relationship between phosphorescence (delay time of 1 ms) and the oxygen activation performance of CDs. (**a**) Fluorescence and phosphorescence (in PVA matrix) spectra of four types of CDs. Inset shows the solution of CDs at different temperatures of synthesis. (**b**) ^1^O_2_ phosphorescence emission spectrum of the four types of CDs in a CD_3_CN−D_2_O mixed solvent (*v/v* = 15/1). (**c**) TMB photo-oxidation efficiencies of the four CDs. Reproduced with permission from Ref. [43]. Copyright © 2022 *ACS Applied Materials & Interfaces*, American Chemical Society.

Optimizing the photophysical characteristics of CDs molecules has been an essential strategy to improve the efficiency of PACT, as seen by increased fluorescence and phosphorescence quantum yields. Wu et al. used citric acid and ethylenediamine as raw ingredients to make a variety of nitrogen-doped carbon dots (CDs). The phosphorescent quantum yield was positively associated with the CDs’ photosensitization ability. From the standpoint of material design, a better photosensitizer can be obtained from the cross between systems from an excited singlet state to an excited triplet state (Figure 4). When the designed CDs were applied to photodynamic antibiotics, the inhibition rates of *Salmonella* and *E. coli* (92% and 86%) were much higher than the growth inhibition rates of phloxine B (40% and 55%), demonstrating the excellent photodynamic antibacterial effect of these CDs [43].

In addition, using the “heavy atom effect” can also be demonstrated to improve the advantages of intersystem crossing (ISC), leading to a phosphorescent deactivation process, energy and electron transfer and more effectively antibacterial PSs [90]. The work of Knoblauch et al. showed that the intersystem crossing could be optimized by doping bromine on the CDs produced [91]. The “heavy atom effect,” which involves incorporating elements such as bromine into tiny molecules, has long been an approach for obtaining better phosphorescence from fluorophores [92]. ROS generation benefits from the triplet interaction between the triplet-excited state and molecular oxygen at the ground state. It has been demonstrated that adding bromine to CDs can result in excellent spin-orbit coupling and subsequent phosphorescence detection. Under UV-A irradiation, the prepared brominated carbon dots (BrCDs) produced HO^•^ (Type I) and ^1^O_2_ (Type II) and displayed considerable antibacterial activity. The potential of hydrogen-departing reactive nitrogen species was discovered in the synthesized CDs, which impeded colony formation even without photodynamic processes [91].

The surface charge of CDs has a significant impact on their PACT effect. Yang et al. performed three studies in which they prepared CDs and then surface functionalized them with 2,2-(ethylenedioxy)bis(ethylamine) (EDA) and 3-ethoxypropylamine (EPA) to produce EDA-CDs and EPA-CDs, respectively [93]. EDA is protonated and becomes positively charged at the neutral potential of hydrogen, but EPA is not. The researchers discovered that EDA-CDs have considerably stronger antibacterial activity than EPA-CDs, implying that the positive charge on EDA-CDs can aid CDs to electrostatically attach a negatively charged bacterial film, improving the PACT effect. According to the authors, a high fluorescence quantum yield is also favorable to the PACT effect. Furthermore, the antibacterial activity of CDs is enhanced by a thin polymer passivation layer.

It is also required to prepare CDs with targeting ability to optimize the photodynamic effect of CDs. Galan et al. found that green fluorescent FCDs made by microwave using glucosamine hydrochloride and m-phenylenediamine as precursors can label *E. coli*, *Klebsiella pneumoniae*, *Pseudomonas aeruginosa*, and Gram-positive (*S. aureus*) pathogens in less than 10 min. FCDs paired with LED irradiation successfully kill Gram-negative and Gram-positive bacteria in the visible range [94].

## 4. Carbon Dots-Based Nanocomposites in PACT

While basic research into the mechanism and optimization of CDs as photosensitizers is still ongoing, researchers have begun to investigate how these particles might be integrated into hybrid systems to improve antimicrobial efficiency. In other cases, the CDs’ intrinsic antibacterial capabilities have been exploited to increase the overall system’s efficiency.

### 4.1. Antibiotic-Modified Carbon Dots

CDs not only have high antibacterial and antimicrobial membrane activity, but they also include a variety of hydrophilic groups on the surface, including -NH_2_, -OH, and -COOH, and their unique nanostructure allows them to be used in many drug delivery applications. The higher cellular internalization of these CDs is used to boost drug absorption. At the same time, it can bring active molecules attached to the main structure by functionalization groups. This property has been discovered to be directly employed in the administration of antibiotic molecules such as vancomycin [95], ampicillin [96], penicillin [97], ciprofloxacin [98,99], and antiparasitic creams [97] to destroy microorganisms in bacterial cells. Bacteria can be killed more effectively by a synergistic combination of the PACT action with CDs antibiotics.

**Figure 5:**
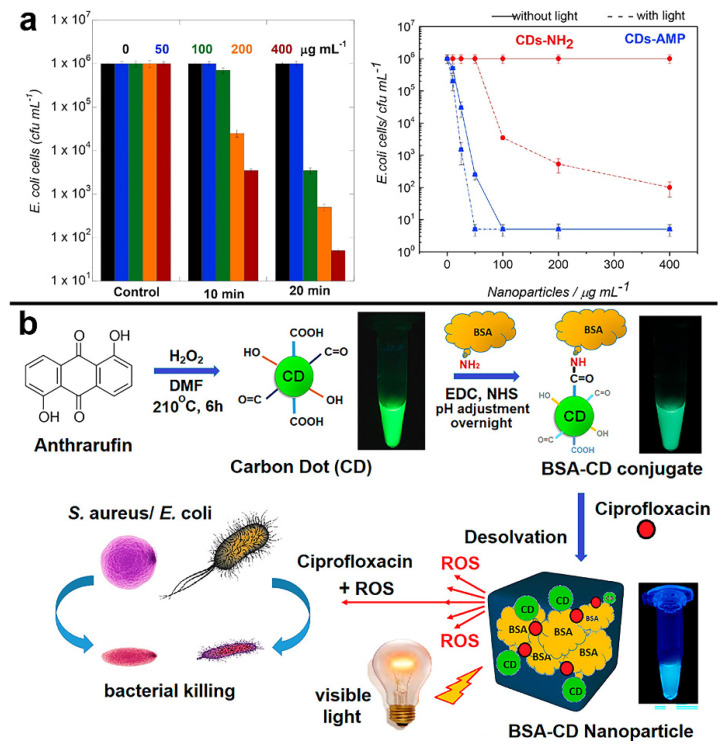
(**a**) Photodynamic efficiency of CDs-NH_2_ for the inactivation of *E. coli* K12-MG 1655 upon irradiation at 0.30 W for 10 and 20 min and influence of the CDs-NH_2_ and CDs-AMP concentration on the treatment efficiency of *E. coli* without (solid lines) and with (dash lines) visible light illumination (20 min, 0.30 W). Reproduced with permission from Ref. [96]. Copyright © 2018 *Colloids and Surfaces B: Biointerfaces*, Elsevier B.V. (**b**) Scheme for Synthesis of CDs, conjugation of the CDs to BSA, and subsequent creation of BSA-CDs nanoparticles for visible-light-induced ROS generation and simultaneous release of ciprofloxacin for antibacterial activity. Reproduced with permission from Ref. [100]. Copyright © 2022 *ACS Applied Materials & Interfaces*, American Chemical Society.

Boukherrub et al. used a hydrothermal approach to prepare amine-functionalized carbon dots (CDs-NH_2_) using citric acid and ethylenediamine as primary materials. To make CDs-AMP nanostructures, the main amine groups on the surface of CDs-NH_2_ were employed to be covalently connected to ampicillin (AMP), a classical β-lactam antibiotic. The lowest dose of CDs-AMP conjugate(14 g mL^−1^)-inhibited *E. coli* cells was higher than free AMP (25 g mL^−1^), confirming the superiority of the CDs-AMP conjugate. As shown in Figure 5a, the authors also confirmed that exposing CDs-AMP to visible light irradiation increased its bactericidal action. When compared to free AMP, the results of this investigation demonstrated that AMP placed on CDs had improved stability and antibacterial activity when exposed to visible light [96].

Mandal et al. used a solvothermal approach to make 1,5-dihydroxyanthraquinone-based CDs that emit green fluorescence. BSA was coated on the surface of the CDs via an amidation reaction to boost ROS activity. As given in Figure 5b, Ciprofloxacin reacted non-covalently with BSA-CDs conjugates to generate drug nanocomplexes. At the same time, PACT functioned synergistically with antibiotic drug release to kill 95% of *E. coli* and *S. aureus* at concentrations as low as 1.47 g mL^−1^ in their complexes [100].

Using CDs conjugated with antibiotics can improve their antimicrobial activity in many cases. Combining photodynamic sterilization with antibiotic sterilization can maximize bacteria-killing because CDs improve the internalization efficiency and targeting of the material and have a photodynamic effect [96].

### 4.2. Carbon Dots as Nanocarriers for Photosensitizers

PSs such as MB and curcumin can also be effectively bound to CDs through covalent coupling or supramolecular interactions [101] (including π–π stacking, electrostatic interactions, etc.) using the same rational approaches to the development of CDs-attached antibiotics.

CDs’ intrinsic antibacterial properties can sometimes boost antimicrobial efficacy. In recent years, mixing CDs with photosensitizers molecules has been discovered to boost overall antimicrobial activity. Dong et al. for example increased the antibacterial action of CDs by combining them with the photosensitizers MB and toluidine blue (TB). According to the scientists, CDs (5 g mL^−1^) alone had no antibacterial effect, and MB (1 g mL^−1^) had little antimicrobial activity, but mixing CDs (5 g mL^−1^) with MB (1 g mL^−1^) considerably increased their antimicrobial effect and almost entirely inhibited bacterial growth, according to the scientists. In *E. coli* cells, the combination of CDs and TB showed a similar synergistic impact. The authors speculate that this is due to (1) increased cellular penetration by small-molecule photosensitizers, (2) improved solubility of their small-molecule counterparts by CDs and thus improved uptake/localization and target delivery, or (3) increased overall intracellular ROS by the combination of both photosensitizers, for example through a fluorescence resonance energy transfer (FRET) mechanism [25].

**Figure 6:**
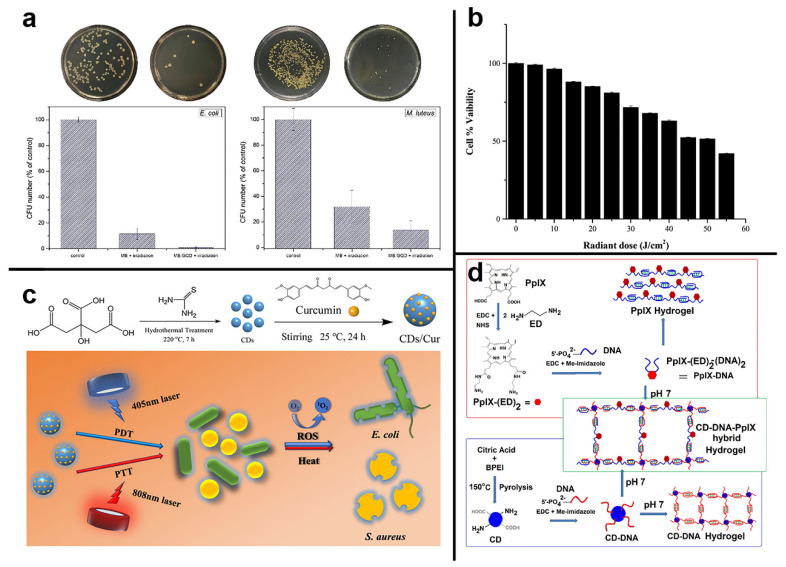
(**a**) (left) *E. coli* (5 min irradiation) and (right) M. Luteus colonies (2 min irradiation) in PBS before and after irradiation and their percentage irradiation of MBGQD with 660 nm light. Reproduced with permission from Ref. [102]. Copyright 2018 *Photodiagnosis and Photodynamic Therapy*, Elsevier B.V. (**b**) Effects of optimized blue light and optimized cur-GQD concentration on NIH/3t3 cells, indicating nontoxic effect of light dose at 30 J cm^−2^ with cur-GQD. Reproduced with permission from Ref. [103]. Copyright © 1999–2022 *Photochemistry and Photobiology*, John Wiley & Sons. (**c**) Synthesis of the CDs/Cur nanocomposite photosensitizer, and bactericidal activities of CDs/Cur upon dual-wavelength (405 + 808 nm) illumination. Reproduced with permission from Ref. [104]. Copyright 2022 *ACS Applied Bio Materials*, American Chemical Society. (**d**) Scheme for conjugation of cytosine, rich single-stranded DNA to CDs and PpIX for hydrogel formation. The blue and red color-coded DNA sequence is the same. Reproduced with permission from Ref. [105]. Copyright © 2019 *Journal of Colloid and Interface Science*, Elsevier Inc.

Similarly, Kholikov et al. synthesized biocompatible and photostable GQDs that produced more monoclinic oxygen when mixed with MB. The population of excited triplet-state photosensitizers created by the inter-systemic crossover (ISC) of excited singlet-state photosensitizers is related to the oxygen generation efficiency of singlet photosensitizers. As a result, GQDs may lengthen the duration of the MB triplet state, boosting (ISC) efficiency from the singlet excited state to the triplet excited state of MB. Within 5 min of irradiation with visible light at the appropriate wavelength, Gram-positive and Gram-negative bacteria treated with the MB-GQD were inactivated (Figure 6a). The lower doses of MB provided higher antimicrobial activity when sulfur-doped GQDs were combined with MB [102]. Yameen et al. synthesized a compound (cur-GQDs) by loading curcumin onto GQDs to increase the solubility and biocompatibility of curcumin. It was shown that loading curcumin onto GQDs solved the problem of curcumin’s poor water solubility and increased its ROS production by three-fold, effectively being an inhibitory effect on *Pseudomonas aeruginosa*, MRSA, *E. coli* and *Candida albicans.* The results were observed when the samples were exposed to 405 nm blue light at 30 J cm^−2^. As shown in Figure 6b, *Pseudomonas aeruginosa*, MRSA, *E. coli* and *Candida albicans* were significantly inhibited [103].

Other types of CDs have excellent photothermal properties but are not directly associated with photodynamic properties. This material class could be under near-infrared light, transforming light energy into heat, and inducing desaturation of enzymes on bacteria’s surfaces by raising their temperature and destroying the cell membrane and biofilms framework. Based on this foundation, a nanocomposite system with synergistic PDT and PTT therapy can be built. The antimicrobial effect can be considerably increased by mixing CDs with photosensitizers molecules via FRET. Su et al. developed a carbon dot composite system CDs/Cur that uses CDs as a carrier for curcumin, which improves curcumin biocompatibility and ROS yield and has an excellent photothermal impact. CDs/Cur may create both heat and generate ROS under dual-wavelength irradiation at 405 and 808 nm, enhancing the antibacterial efficacy by combining PDT/PTT (Figure 6c). The killing effect on *E. coli* was up to 100% of the final concentration of 1 M CDs/Cur concentration. In contrast, the lethal effect on *S. aureus* was even stronger, with a mortality rate of 100% at the lowest concentration of 0.10 nM [104].

CDs have excellent fluorescence properties, including broad-spectrum absorption, tunable photothermal effects, upconversion luminescence, and visible light absorption, allowing them to be used as FRET donors in various applications. Traditional photosensitizers such as PpIX have low solubility and are prone to aggregation-induced bursts. Das et al. developed the CD-DNA-PpIX hybrid hydrogel using protoporphyrin as the acceptor. DNA functions as a linker to join CDs and PpIX, combining the preceding advantages of CDs. As shown in Figure 6d, CDs serve two purposes: a cross-linker to disseminate PpIX and a FRET donor to stimulate PpIX. The photodynamic effect of PpIX in visible light and CDs to produce FRET can work together to generate additional ROS, which can considerably improve the photodynamic outcome. The length of the DNA’s sequence impacts the distance between the CDs and PpIX, as well as the efficiency of FRET, which can minimize PpIX’s self-burst and ensure its delayed release. The hydrogel was entirely over at 10–11 days in the experiment, while PpIX produced ROS slowly and consistently, killing Gram-positive bacteria (*S. aureus*) continually [105].

### 4.3. Carbon Dots/Metal Oxide Nanocomposites

CDs/metal oxide nanocomposites, such as ZnO/GQDs [106], CDs/Na_2_W_4_O_13_/WO_3_ [107], CDs/TiO_2_ [108] and CDs/Cu_2_O [109], are also under evaluation for its antimicrobial activity. When these materials are exposed to UV–visible light, these nanocomposites release ROS, which kill microorganisms by some previous mechanisms presented. For example, Chen et al. used a hydrothermal technique to make ZnO/GQDs nanocomposites that could form reactive oxygen species to kill *E. coli* when exposed to UV light. Their bactericidal activity was much higher than ZnO and GQDs [106].

### 4.4. Other Hybrid Carbon Dots

**Figure 7:**
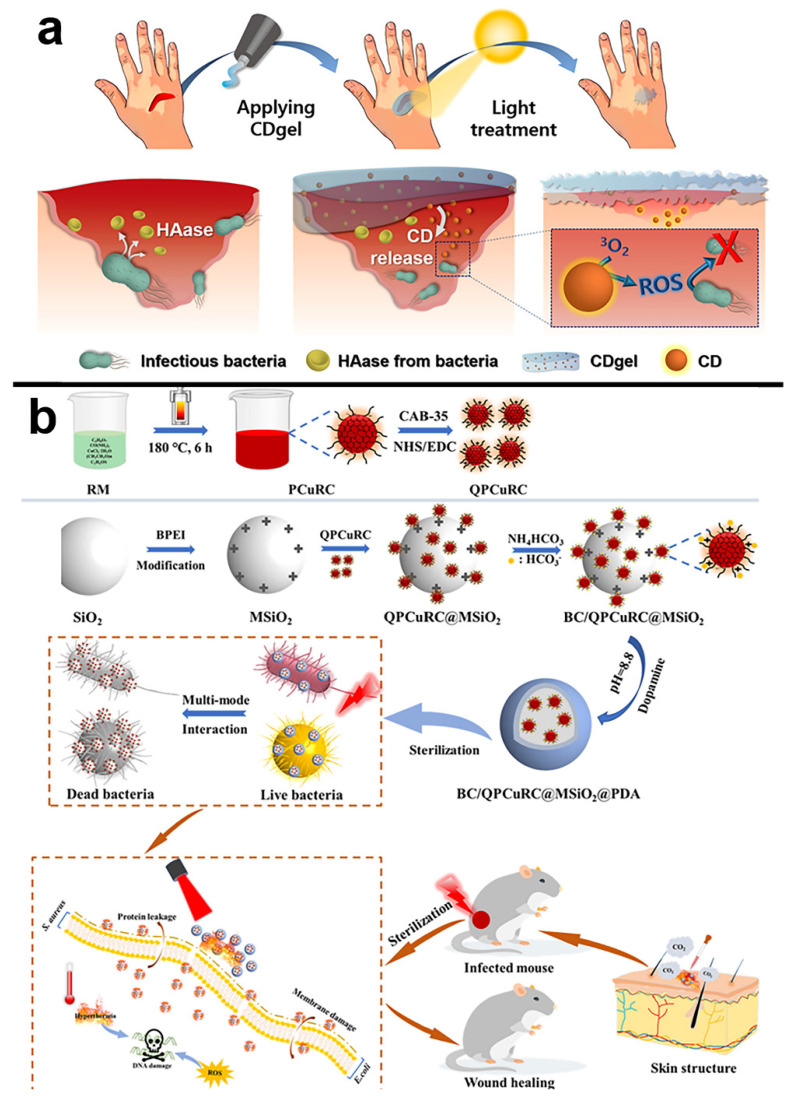
(**a**) Diagram of a CDs-embedded hyaluronic acid-based hydrogel (CDgel) for photoablation of infectious bacteria. Infectious bacteria such as *S. aureus* and *E. coli* produce hyaluronidases (HAase) when they increase. The CD gel’s hyaluronic acid is decomposed by hyaluronidases, resulting in the release of the CDs. The released CDs use ^1^O_2_ production to photodynamically ablate infectious bacteria, allowing for the selective and targeted removal of malignant germs from infected wounds. Reproduced with permission from Ref. [110]. Copyright © 2022 *ACS Applied Bio Materials*, American Chemical Society. (**b**) Schematic illustration of the synthesis of BC/QPCuRC@MSiO_2_@PDA and their related biological applications. Reproduced with permission from Ref. [111]. Copyright © 2021 *Journal of Colloid and Interface Science*, Elsevier Inc.

Many experiments have been conducted to improve the antibacterial photodynamic properties of CDs. The embedding of light-responsive CDs into soft hyaluronic acid hydrogels is frequently used as a photoactive antibacterial technique. Infectious bacteria in the target tissue can dissolve hydrogels structure and liberate CDs because of the action of hyaluronidase present on infectious bacteria naturally. Park et al. created a light-responsive carbon dot-embedding soft hyaluronic acid hydrogel (CDgel) to be used as a photodynamic antibacterial agent in vivo and in vitro by embedding CDs into a hyaluronic acid cross-linked hydrogel. As previously stated, the hyaluronic acid backbone of CDgel is broken by the bacterial hyaluronidase enzyme when applied to the bacterial site. CDgel degrades at this moment, transitioning from a gel to a liquid state, and CDs are released as a result. CDs release vast levels of ^1^O_2_ when exposed to white LED light, which can kill up to 99% of *E. coli* and 97% of *S. aureus* (Figure 7a) [110].

In addition, the development of a synergistic antibacterial platform can also greatly enhance the antibacterial performance. As shown in Figure 7b, Shen et al. developed silicon-based near-infrared CDs (QPCuRC@MSiO_2_) and a bicarbonate (BC) nanoplatform (BC/QPCuRC@MSiO_2_@PDA). It has triple synergistic antibacterial properties such as PDT, PTT and quaternary ammonium compounds (QACs). In vitro and in vivo experiments showed that BC/QPCuRC@MSiO_2_@PDA had excellent antibacterial properties, and the antibacterial rates against *S. aureus* and *E. coli* could reach 99.99% and 99.60%, respectively [111].

The application of carbon dots-based nanocomposites in PACT is summarized in this section. To boost the effect of PACT, CDs can be employed as an antibiotic carrier, mixed with a photosensitizer, combined with a metal oxide, or formed into various hybrid materials.

In Table 1, we have listed some representative CDs structure presenting high potential activity on PACT, based on the material discussed in Section 3 and Section 4.

## 5. Toxicology and Safety Profile of Carbon Dots

CDs generally have good cytotoxicity and biocompatibility, which is beneficial for biomedical applications. However, the influence of CDs on host cells must be carefully evaluated before CDs can be widely used in PACT applications, and its possible medical complications and detailed toxicology need to be further studied. The toxicity of CDs is particularly important because of the unavoidable contact between mammalian cells or tissues and photosensitizers during PACT application. Additionally, photosensitizers are prone to transfer from the surface to the biological system, resulting in potential safety risks.

Thus far, much work has been performed on the cytotoxicity of CDs on mammalian cells, and it has been reported that they are non-toxic at proper concentrations both in vitro and in vivo [112,113,114]. However, the cytotoxicity of the CDs should not be overlooked because the properties of CDs vary greatly between different precursors and synthetic strategies. Although CDs prepared from “bio-safe” precursors such as common glucose are commonly believed to be nontoxic, sometimes they maintain low cytotoxicity only in dark conditions, while cytotoxic substances could be produced upon light irradiation. Therefore, extensive studies must be carried out to fully explore the possible toxic effects of CDs on humans. The used concentration of CDs is also an important factor affecting its toxicity. A high concentration of CDs will exert toxic effects on the central nervous system. Toxicology reports of GQDs indicate that although most existing studies support the safe use of GQDs, their toxicity may vary depending on the concentration and test method used in the synthesis technology. Studies have found that small-sized CDs are more toxic than large-sized CDs [115], and CDs with negative charged are more cytotoxic to mammalian cells [116,117]. ROS production plays an important role in the sterilization process of CDs, but ROS may also cause cell death. In order to solve the above problems, it is necessary to promote safe and controllable CDs synthesis strategies and application methods, and the safe application of CDs in the treatment of infectious diseases requires in-depth research on its possible toxic side effects and complications.

## 6. Conclusions

As presented, CDs have received much attention in chemical sensing, biological imaging, photocatalysis, phototherapy, and drug administration. The CDs’ structure work as a photosensitizer in PACT is discussed in many aspects and applications in this paper. The synthetic process for CDs preparation was evaluated first to propose classical and new routes for synthesizing CDs with high photodynamic antibacterial effects. Then, the key elements that could be impacting the antibacterial effect of CDs were also discussed. Furthermore, more effective antibacterial materials can be created by mixing CDs with other photosensitizers molecules and antibiotics or by creating hybrid materials based on CDs. CDs have been shown to be one of the most promising carbon classes of material to work properly as an antibacterial material because of their excellent physical and chemical properties, optical qualities, and photophysical and photochemical behavior associated with exceptional water solubility.

Conversely, CDs face some problems, limiting their practical application. The exact process of photoluminescence is unknown, and CDs with extended excitation and emission wavelengths are still uncommon, leading to complex tissue and biofilm penetration. Second, relatively few CDs have intrinsic microbe targeting ability, resulting in a significantly reduced antibacterial effect that is essential in developing antibacterial CDs. Finally, CDs’ water solubility and biocompatibility influence their microbial therapy usage. To summarize, the creation of high-efficiency antibacterial CDs faces numerous hurdles. As these issues are resolved, CDs may have more good results in microbial therapeutics.

## Figures and Tables

**Table 1:** Representative CDs for killing microorganisms; from Section 3 and Section 4.

CDs Label ^a^	The Precursor of CDs	Excitation Wavelength	Emission Wavelength	QY	Light Wavelength	Light Power	ROS Sensitization Yields	Microorganism	Reduction of Bacteria	Ref.
GQD	graphite rods	328 nm	494 nm	--	blue light (470 nm)	1 W	--	*S. aureus and E. coli*	80% *E. coli* and 90–95% *S. aureu* were eliminated after 15 min *	[76]
MCDs	edible mushroom	360 nm	456 nm	25%	visible LED light	2.70 mW cm^−2^	--	*E. coli*	>90% elimination of *E. coli* in 12 h	[78]
CDs	citric acid	370 nm	450 nm	--	blue light (450 nm)	40 J cm^−2^	--	*S. aureus*	total elimination of *S. aureus* suspension was achieved (CDs: 6.90 mg/mL) and total elimination of the biofilm cultures was achieved (CDs: 13.80 mg/mL)	[80]
GQD	graphite	480/740 nm	618–647 nm	18.50%	800 nm	2.64 mW	QY = 0.51 (^1^O_2_)	*E. coli and* MRSA	all *E. coli and* MRSA to be dead after the 15 s laser photoexcitation	[83]
Cur-NRCDs	curcumin, neutral red and citric acid	540 nm	635 nm	--	xenon light (400–450 nm)	--	--	*S. aureus and E. coli*	after 10 min of xenon irradiation, 10 mM and 15 mM of Cur-NRCDs can kill 100% of *S. aureus* and *E. coli*, respectively	[88]
N-GQD (5.1%)	graphite	365 nm	624 nm	25.90%	670 nm laser	0.10 W cm^−2^	QY = 0.64 (^1^O_2_)	*E. coli*	100% was eliminated by N-GQDs (5.1%) after a 3-min exposure	[89]
CDs	citric acid and ethylenediamine	350 nm *	450 nm	20%	LED light (365 nm)	3 V/3 W.	QY = 0.82 (^1^O_2_)	*E. coli and Salmonella*	bacteria growth inhibition efficiencies of 92% and 86% were obtained for *E. coli* and *Salmonella* in the presence of 5 μM CDs with light in 1 h, respectively	[43]
BrCDs	natural gas, HBr	302 nm	>355 nm	--	Ultraviolet lamp (365 nm)	3 mW	--	*Listeria monocytogenes, S. aureus and E. coli.*	with 10 min of UV exposure the growth of each bacterium is further decreased, achieving minimal to no colony formation visible for each	[91]
EDA-CDs/EPA-CDs	carbon nano-powders	--	--	20%	400–800 nm light bulb	36 W, 12 V	--	*Bacillus subtilis*	1 h of EDA-CDs and EPA-CDs treatment resulted in a reduction of approximately 5.80 log and 0.84 log, respectively	[93]
FCDs	glucosamine hydrochloride and m-phenylenediamine	--	--	--	blue-LED strip lights (460 nm)	24 W, 12 V	--	*Klebsiella pneumoniae, Pseudomonas aeruginosa, E. coli and S. aureus*	complete killing of each bacterium was reproducibly observed after treatment with 200 µg/mL FCDs with 4 h of irradiation, and significant killing (>95%) could be observed after only 90 min LED irradiation	[94]
**Antibiotic-Modified CDs**										
CDs-AMP	citric acid and ethylenediamine	350 nm	450 nm	19%	visible light	0.30 W	--	*E. coli*	>4 log_10_ inhibition of *E. coli* by CDs-AMP after 20 min of irradiation *	[96]
BSA-CDs NP	1,5-dihydroxyanthraquinone	395 nm	525 nm	75% (CDs)	Tungsten bulb (300–900 nm)	100 W	--	*S. aureus and E. coli*	99.97% and 99.53% elimination of *E. coli* and *S. aureus* in 1h	[100]
**CDs as nanocarriers for photosensitizers**										
CDs/MB or CDs/TB	carbon nanopowders	400 nm	--	12% (CDs)	white light bulb	36 W	--	*E. coli*	5 μg/mL CDs combined with 1 μg/mL MB completely inhibited bacteria growth, resulting in 6.20 log viable cell number reduction	[25]
GQDs	sulfur and nickel (II) oxide powder and benzene	310 nm	420 nm	--	660 nm red light	12 W	--	*E. coli and Micrococcus luteus*	10^6^ CFU/mL *E. coli* and *Micrococcus luteus* can be eradicated entirely in 10 min with MB-GQD irradiation	[102]
cur-GQDs	coal and curcumin	407 nm	525–550 nm *	--	405 nm LEDs	30 J cm^−2^.	--	*Pseudomonas aeruginosa,* MRSA, *E. coli and Candida albicans.*	for *S. aureus Pseudomonas aeruginosa*, MRSA, *E. coli* and *Candida albicans*, cur-GQDs caused 5.68 log_10_, 5.02 log_10_, 5.44 log_10_, 2.26 log_10_ and 3.82 log_10_ CFU reduction, respectively	[103]
CDs/Cur	citric acid and thiourea	420 nm	550–575 nm*	--	405 + 808 nm light	808 nm (500 mW cm^−2^), 405 nm (200 mW cm^−2^)	--	*E. coli and S. aureus*	death rate of *E. coli* and *S. aureus* increased to 100% for 1 μM and 0.1 nM CD/Cur, respectively	[104]
CD-DNA-PpIX hybrid hydrogel	citric acid and Branched Polyethylenimine	350 nm	625–650 nm *	--	UV lamp (302 nm)	--	--	*S. aureus*	UV irradiation for 2.50 min followed by incubation for 24 h affected > 4.50 log (>99.99%) reduction of *S. aureus* cells	[105]
**CDs/metal oxide nanocomposites**										
ZnO/GQDs	citric acid	365 nm	460 nm	--	UV light (365nm)	100 W, 1000–1500 lumen	--	*E. coli*	100% was eliminated by ZnO/GQDs after 5 min of UV exposure	[106]
**Other hybrid CDs**										
CDgel	ammonium citrate and polyethylenimine	390 nm	400–500 nm	--	white light irradiation	5 mW cm^−2^	--	*S. aureus and E. coli*	CDgel under light giving approximately 99% and 97% mortality for *S. aureus* and *E. coli*, respectively	[110]
BC/ QPCuRC@MSiO_2_@PDA	Citric, urea and CuCl_2_·2H_2_O	360 nm	722 nm	--	808 nm	2 W cm^−2^	--	*S. aureus and E. coli*	antibacterial rate up to 99.60% and 99.99% to *E. coli* and *S. aureus*, respectively	[111]

^a^ Labels indicate either additional details regarding the nature of the reported carbon dots or indicate the abbreviation/common label used within the cited study to describe the particle. * Denotes values extrapolated from relevant in-text details from the specified reference.

## Data Availability

Data sharing not applicable.
